# Nitrogen use efficiency and nitrous oxide emissions from five UK fertilised grasslands

**DOI:** 10.1016/j.scitotenv.2019.01.082

**Published:** 2019-04-15

**Authors:** L.M. Cardenas, A. Bhogal, D.R. Chadwick, K. McGeough, T. Misselbrook, R.M. Rees, R.E. Thorman, C.J. Watson, J.R. Williams, K.A. Smith, S. Calvet

**Affiliations:** a Rothamsted Research, Okehampton, Devon, EX20 2SB, UK; b ADAS Boxworth, Battlegate Road, Boxworth, Cambridge CB23 4NN, UK; c School of Natural Sciences, Bangor University, Bangor LL57 2UW, UK; d Agri-Food and Biosciences Institute, 18a, Newforge Lane, BT9 5PX Belfast, UK; e Scotland's Rural College (SRUC), King's Buildings, West Mains Road, Edinburgh EH9 3JG, UK; f School of Geosciences, University of Edinburgh, Crew Building, Alexander Crum Brown Road, Edinburgh EH9 3FF, and Weston Road, Totnes TQ9 5AH, Devon, UK; g Universitat Politècnica de València, Institute of Animal Science and Technology, Camino de Vera s.n., 46022, Valencia, Spain

**Keywords:** Synthetic fertiliser, Yield scaled N_2_O emissions, Intensification, Nitrogen use efficiency, Emission factor

## Abstract

Intensification of grasslands is necessary to meet the increasing demand of livestock products. The application of nitrogen (N) on grasslands affects the N balance therefore the nitrogen use efficiency (NUE). Emissions of nitrous oxide (N_2_O) are produced due to N fertilisation and low NUE. These emissions depend on the type and rates of N applied. In this study we have compiled data from 5 UK N fertilised grassland sites (Crichton, Drayton, North Wyke, Hillsborough and Pwllpeiran) covering a range of soil types and climates. The experiments evaluated the effect of increasing rates of inorganic N fertiliser provided as ammonium nitrate (AN) or calcium ammonium nitrate (CAN). The following fertiliser strategies were also explored for a rate of 320 kg N ha^−1^: using the nitrification inhibitor dicyandiamide (DCD), changing to urea as an N source and splitting fertiliser applications. We measured N_2_O emissions for a full year in each experiment, as well as soil mineral N, climate data, pasture yield and N offtake. N_2_O emissions were greater at Crichton and North Wyke whereas Drayton, Hillsborough and Pwllpeiran had the smallest emissions. The resulting average emission factor (EF) of 1.12% total N applied showed a range of values for all the sites between 0.6 and 2.08%. NUE depended on the site and for an application rate of 320 kg N ha^−1^, N surplus was on average higher than 80 kg N ha^−1^, which is proposed as a maximum by the EU Nitrogen Expert Panel. N_2_O emissions tended to be lower when urea was applied instead of AN or CAN, and were particularly reduced when using urea with DCD. Finally, correlations between the factors studied showed that total N input was related to Nofftake and Nexcess; while cumulative emissions and EF were related to yield scaled emissions.

## Highlights

• N_2_O emissions and NUE were measured at 5 UK grassland sites.
• Different fertilisation rates and strategies were tested in all sites.
• Average N_2_O emission factor was 1.12%, but ranged from 0.60% to 2.08%.
• Using urea and urea with DCD reduced N_2_O emission factor.
• Yield scaled emissions and emissions relative to herbage N content show similar trend.

## Graphical abstract

**Figure:**
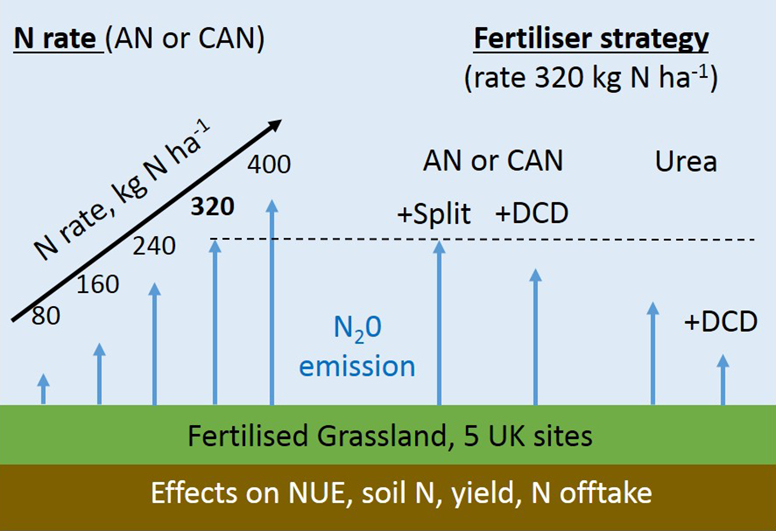

## 1 Introduction

During recent decades, the demand for global food has increased rapidly as a consequence of population growth and changes in patterns of food consumption. One of the most relevant changes in the global agro-food system has been the intensification of production systems and the increase of nitrogen (N) use and trades (Lassaletta et al., 2016). Cultivated grasslands are an example of this intensification process and constitute a significant share of the agricultural area in some temperate countries (FAOSTAT, 2018). It is expected that further intensification will occur to fulfil increasing global demand for livestock products, putting pressure on farming activities that will likely result in increased N use.

N fertilisation of grasslands has relevant productive and environmental effects. It has major effects on the nutritive value of fresh herbage, as well as on animal nutrition and N balance (Lee, 2018). However, fertiliser rates exceeding crop requirements lead to an N surplus, reduced N use efficiency (NUE) and losses to the environment (Van Eerd et al., 2018). In terms of gaseous pollutants, N fertiliser applications are associated with emissions of nitrous oxide (N_2_O) (Reay et al., 2012), a powerful greenhouse gas (GHG) with a large global warming potential (Forster et al., 2007), and a gas that contributes to ozone (O_3_) depletion in the stratosphere (Ravishankara et al., 2009). In the case of urea-based fertiliser applications, ammonia (NH_3_) is also emitted (Pan et al., 2016), with NH_3_ emissions directly implicated in detrimental environmental quality (Krupa, 2003). An improved NUE is required in intensively managed grasslands to reduce the negative effects of an N surplus while preserving productivity and soil fertility.

Nitrogen (organic and manufactured) fertiliser application to agricultural soils is the main contributor to anthropogenic N_2_O emissions, with a share of approximately 60% of the global total (Reay et al., 2012). The Intergovernmental Panel on Climate Change (IPCC) default N_2_O emission factor (EF) is 1% of the applied N (IPCC, 2006). However, recent evidence has demonstrated the complexity of N_2_O emissions in terms of their spatial and temporal variability and the influence of a range of controlling factors, highlighting the need for research to develop system-specific EFs (Butterbach-Bahl et al., 2013).

The emissions of N_2_O are a result of biochemical processes (forms of nitrification and denitrification) in the soil (Hu et al., 2015). The main drivers of these microbial processes are soil moisture (Wu et al., 2017), soil texture, nutrient availability and form, and vegetation (Abalos et al., 2017), which are affected by environmental conditions and land use (Oertel et al., 2016). Management also influences emissions, particularly the form of N fertiliser or manure applied and method of application, soil management (surface application or incorporation), presence of livestock, and the long term effects of fertilisers on soil nutrient status (Cui et al., 2016; LaHue et al., 2016).

The type and rate of N fertiliser has been demonstrated to play a fundamental role in the NUE and N_2_O emissions from grasslands. Rate (Bell et al., 2016) and type of fertiliser, for example calcium ammonium nitrate vs. urea (Harty et al., 2016), use of nitrification inhibitors (NIs) (Gilsanz et al., 2016); split fertiliser applications (Bell et al., 2016), and method of application (Clayton et al., 1997) have also been investigated as potential mitigation options.

**Fig. 1:**
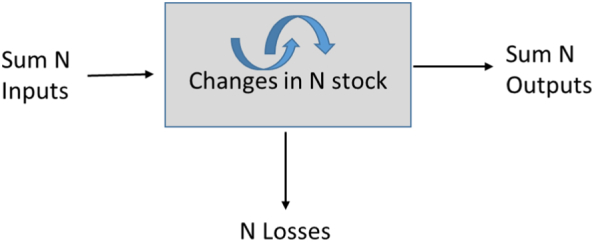
Modified NUE model from the EU N Expert panel. Sum N inputs is Fertiliser N applied + N deposition + N fixation (negligible); N outputs is N offtake; N losses is the difference between N inputs and N outputs.

Nitrogen use efficiency has been suggested as an indicator (or metric) of the N efficiency of agricultural systems by the EU Nitrogen Expert Panel (2015). Fig. 1 shows an adapted version of the Panel's model describing the sum of inputs/outputs and N losses, and changes in N stock. Garnett and Godfray (2012) stated that it is advantageous to depart from the usual crude metrics of sustainability, such as yield, calories or income. They recommend that metrics should allow understanding of dependencies between different farming elements to be able to relate them to anthropometric indicators of health. Common metrics used for gaseous losses, particularly N_2_O, refer to yield (Hinton et al., 2015); we propose to use an additional metric, such as N content in the plant (proxy for crude protein) that reflects more directly what will eventually go to animal and human consumption.

The main objective of this work was to use the data from five 12-month, plot-scale field experiments (details of only one of these experiments were published previously in Bell et al., 2016) to provide evidence on how to improve the use of manufactured N fertiliser and to reduce N_2_O emissions from temperate grasslands. Specific aims included, i) to determine how yield, NUE and N_2_O emissions were affected by increasing rates of ammonium nitrate (AN) fertiliser, in different soil and climatic conditions, ii) to explore how NUE and N_2_O emissions were affected by different N fertiliser mitigation strategies (adding a nitrification inhibitor, splitting applications or replacing AN fertiliser with urea fertiliser, with or without a nitrification inhibitor), and iii) to assess the variability of N_2_O emissions in 5 UK grassland sites and estimate site specific EFs for direct N_2_O emissions following manufactured N fertiliser application. More information related to these experiments is found in www.ghgplatform.org.uk. Archived data sources are: Bell et al. (2017); Cardenas et al. (2017); McGeough et al. (2017); Thorman et al., 2017a, Thorman et al., 2017b.

## 2 Materials and methods

### 2.1 Description of field sites

The experiments were established on five long-term permanent UK grassland sites: Crichton in Scotland, Drayton and North Wyke in England, Hillsborough in Northern Ireland and Pwllpeiran in Wales. At all sites the same treatments were tested, including several rates and types of manufactured N fertiliser. The measurement teams followed a joint experimental protocol developed for the GHG platform project, funded by UK Defra and the Devolved Administrations, which has been described previously in Bell et al. (2016). The sites covered a range of locations typical of grassland soils and climate in the UK, as listed in Table 1. Data from the nearest (<1 km) meteorological station were used at each site to provide air temperature and daily rainfall. Additionally, representative soil samples (0–7.5 cm) were taken from each site before the start of the measurements to determine the general soil characteristics. Soil descriptions and dates of experiments are also included in Table 1. The experiments lasted for 12 months, starting in March 2011 (Crichton and Hillsborough), February 2013 (North Wyke) and March 2013 (Drayton and Pwllpeiran). Established grasslands (>5 years old except for Hillsborough, which was 3 years old) with ryegrass (*Lolium perenne*) were used for the experiments. Grazing livestock were excluded from the fields for the 6 months before the measurements started, and also during the experiments. Further details of the experimental design and measurements are described in detail by Bell et al. (2016).

**Table 1:** Location, measurement periods and soil characteristics of the experiments in each site.

	Crichton	Drayton	North Wyke	Hillsborough	Pwllpeiran
Location	Dumfries, Scotland	Warwickshire, England	Devon, England	County Down, Northern Ireland	Ceredigion, Wales
Altitude (m)	50	47	181	128	213
Measurement period	22/03/11–21/03/12	04/03/13–03/03/14	27/02/13–26/02/14	22/03/11–21–03-12	04/03/13–03/03/14
Soil texture	Sandy loam	Clay	Clay loam	Clay loam	Clay loam
Soil pH	5.6	7.6	5.5	6.5	5.5
Soil clay content (%)	15	56.5	41.3	25.7	31
Bulk density (g cm^−3^)	1.12	0.86	0.71	0.74	0.95
Soil organic carbon (%)	3.5	4.7	4.5	9.0	4.7
Soil total nitrogen (%)	0.3	0.6	0.6	0.6	0.5
Initial soil NO_3_^−^ (kg N ha^−1^)	n.a.	1.14	2.49	3.45	3.14
Initial soil NH_4_^+^ (kg N ha^−1^)	n.a.	2.50	1.43	4.90	3.54
Olsen P (mg/L)	13.3	19.5	30.0	39.2	17.0

n.a.: not available.

Initial soil NO_3_^−^ and NH_4_^+^ correspond to the average content of all treatments 3 days before the first application.

### 2.2 Application of treatments

Nine fertiliser treatments and one control (i.e. no N applied) were established, with the first application in early spring, as described in Table 2. Five of the treatments comprised increasing rates of ammonium nitrate (AN) from 80 to 400 kg N ha^−1^, at regular intervals. Calcium ammonium nitrate (CAN) was applied at Hillsborough instead of AN due to different fertiliser regulations in Northern Ireland compared to the rest of the UK. Fertiliser was applied in four split applications following recommendations from the country's fertiliser guide. Application dates and rates are detailed in Table 2. For comparison, additional N fertiliser treatments were also applied at the 320 kg N ha^−1^ application rate. These additional treatments evaluated the effect of substituting AN by urea (U), splitting the two first applications (making a total of six applications, see Table 2) and adding a nitrification inhibitor (dicyandiamide, DCD) to either AN or U. DCD was applied as a 2% solution (i.e. 20 g/1000 mL) at a rate equivalent to 10 kg ha^−1^ DCD (i.e. 500 L ha^−1^ of 2% solution) after each fertiliser application. DCD application rate and method was selected according to commercial guidelines and previous research (Moir et al., 2007). The DCD solution was applied within 1 h after the fertiliser was applied using calibrated spray equipment. As DCD contains 65% N, the N fertiliser applications were reduced to compensate and to ensure that the same amount of plant available N was applied. Also, to prevent any nutrient deficiencies, overall basal P, K and Mg fertilisers and S were applied according to site requirements (RB209, BSFP, 2010). At all sites, there were three replicates of each treatment in a randomized block design. In each block ten plots (treatments) were established. Plot size varied between sites, depending on equipment availability, from 2 to 6 m wide and 12 to 24 m long. Each plot was divided into two areas: one area where soil samples were taken and five N_2_O chambers were placed, and a second area (a minimum of 15 m^2^) which was kept undisturbed for grass yield measurements.

**Table 2:** Fertiliser application rates (kg N ha^−1^), application dates and harvest dates. Numbers in parenthesis indicate the amount of fertiliser on each application date. Underlined dates of application refer to additional applications in the AN320 Split treatment (see text for details). AN is ammonium nitrate; U is urea; CAN is calcium ammonium nitrate.

	Doses and dates
Treatments^a^	
Control	0
AN80	80 (20, 20, 20, 20)
AN160	160 (30, 40, 50, 40)
AN240	240 (40, 60, 80, 60)
AN320	320 (70, 70, 100, 80)
AN400	400 (90, 90, 120, 100)
AN320 + DCD	320 (70, 70, 100, 80)
U320	320 (70, 70, 100, 80)
U320 + DCD	320 (70, 70, 100, 80)
AN320 Split	320 (30, 40, 30, 40, 100, 80)
Application dates	
Crichton (2011–2012)	21/03, 04/04, 15/04, 22/04, 18/05, 04/07
Drayton (2013–2014)	04/03, 18/03, 02/04, 15/04, 28/05, 15/07
North Wyke (2013–2014)	26/02, 11/03, 02/04, 15/04, 28/05, 25/06
Hillsborough (2011–2012)	21/03, 04/04, 18/04, 25/04, 16/05, 04/07
Pwllpeiran (2013–2014)	04/03, 18/03, 02/04, 15/04, 21/05, 12/07
Harvest dates	
Crichton	16/05, 29/06, 17/08
Drayton	22/05, 08/07, 09/09
North Wyke	22/05, 18/06, 23/07, 20/09
Hillsborough	10/05, 27/06, 15/08
Pwllpeiran	19/05, 10/07, 04/09

a CAN was used in Hillsborough instead of AN.

### 2.3 Herbage yield, N offtake and N use efficiency

Grass was harvested three times from each site (four from North Wyke) during the growing season, following the typical practice for a grass-silage system in the UK, at the dates shown in Table 2. Grass yield was measured from the 15 m^2^ undisturbed area on all treatment and control plots. Grass dry matter (DM) content was determined by drying at 85 °C for 24 h (Cardenas et al., 2016). The grass N content was determined on ground dried material using a Carlo Erba NA2000 analyser (CE Instruments, Wigan, UK) and a SerCon 20–22 isotope ratio mass spectrometer (SerCon Ltd., Crewe, UK) at the North Wyke site and similarly at the rest of the sites. Wheat flour (IA-R001 from Iso-Analytical, Crewe, UK; 1.88% N, 40.2% C, 2.55 δ^15^N and −26.43 δ^13^C) calibrated against IAEA-N-1 for nitrogen and IAEA-CH6 for carbon by Iso-Analytical, Crewe, UK was used as a reference standard (Rowell, 1994).

NUE was calculated as defined by the EU Nitrogen Expert Panel (2015). This indicator is based on the mass balance principle, and is calculated as NUE = N output / N input, where N output is the total N measured in the harvested grass, and N input the amount of fertiliser N applied in the year, and atmospheric N deposition; biological N fixation was considered negligible because the major grassland species was ryegrass. N input due to atmospheric deposition was estimated according to the NGAUGE model (Brown et al., 2005) to be 25 kg N ha^−1^ year^−1^ for all sites except for Crichton, where it was 35 kg N ha^−1^ year^−1^. NUE was calculated for all sites at the different N application rates on an annual basis. NUE is presented following the conceptual framework defined by the EU Nitrogen Expert Panel (2015) in which the N fertiliser application rate can be defined by three potential outcomes: (i) an N surplus lower than 80 kg N ha^−1^ to reduce pollution risk; (ii) an NUE lower than 90% to avoid soil mining, and (iii) a desired productivity of at least 80 kg N ha^−1^. To evaluate the performance of the different N applications, NUE was also calculated for the intervals between consecutive fertiliser applications (Phases 1, 2 and 3), as well as between the last fertiliser application and the final harvest (Phase 4). Since intermediate harvest dates did not coincide with the N fertiliser application dates, the accumulated N offtake by the grass was modelled using a non-linear regression model (logistic curve) of GENSTAT (GenStat 14th Edition, Version 14.1., VSN International Ltd., Oxford, UK), and N offtakes at the exact fertiliser application dates were estimated for each site and treatment. All models fitted satisfactorily (P < 0.05, R^2^ > 0.98, details not shown). N losses in the model would include all N gases (N_2_O, N_2_, NH_3_) and NO_3_^−^ leaching.

### 2.4 Measurement of N_2_O fluxes

Nitrous oxide emissions were measured using the closed static chamber method (Chadwick et al., 2014) with further detail explained in Bell et al. (2016). Briefly, at the beginning of the experiment, five opaque chambers were placed in each plot. Chambers were inserted in the soil to a depth of approximately 5 cm to ensure an adequate seal.

Gas sampling was always done between 10:00 to 14:00 to avoid the potential effect of diurnal variation. When conducting the measurements, a lid was placed on top of the chamber for 40 min, and the accumulation of N_2_O inside the chamber was used to calculate the emissions (see Chadwick et al., 2014). To determine N_2_O concentrations, a gas sample was taken using a 50 mL syringe either through a septum in the lid or through a 3-way valve previously placed on the lid. Also, five ambient samples were taken before chambers were closed, and another 5 at the end as representative of the background (T0) N_2_O concentration. Gas samples were transferred to 20 mL pre-evacuated glass vials and transported to the laboratory. The concentration of greenhouse gases was determined by gas chromatography (Perkin Elmer Clarus 500 gas chromatograph fitted with a Turbomatrix 110 automated headspace sampler, and an electron capture detector set at 300 °C for N_2_O analysis) at the North Wyke site. Systems were similar at the other sites and are described in Bell et al. (2016) and McGeough et al. (2016). In addition, to check on the linearity of gas accumulation within a chamber's headspace, on every N_2_O sampling occasion 3 chambers were selected at random from the treatment with the highest N input. From each of these 3 chambers, a time series of samples following closure were taken: 6 samples at spaced intervals of 0, 10, 20, 30, 50, 60 min or 0, 15, 30, 45, 60 min (depending on the site).

Gas sampling was more frequent following each N fertiliser application. One measurement was taken few days before each application as a background value, and then after each application 5 measurements were taken daily for the following two weeks. Sampling was reduced to 2 days per week for the next three weeks, then one sample every two weeks for 5 months followed by once per month until the end of the experiment. This frequency of measurement protocol was started again after each fertiliser application.

Emissions were calculated from the increase of N_2_O concentration and the chamber height, following the ideal gas law. Cumulative annual emissions were determined for each chamber by adding the area under the curve between sampling dates. This assumes a linear change in emissions between two consecutive measurements (Chadwick et al., 2014). For each site, average values and the standard error (s.e.) per treatment were reported. Emission factors for each treatment and block were calculated following the IPCC methodology (IPCC, 2006), i.e. where the cumulative N_2_O-N emitted from the control is subtracted from the cumulative N_2_O-N emitted from the treatment, and the net emission divided by the quantity of N applied in the fertiliser. Yield scaled N_2_O emissions were also calculated for all treatments where the cumulative annual N_2_O-N emissions (kg ha^−1^) were divided by the total (all harvests) grass N offtake.

### 2.5 Other measurements

Soil moisture content was determined for each block on all N_2_O measurement days (70 to 100 times depending on the site). Five soil samples (0–10 cm depth) were collected randomly to obtain a representative sample. Soil moisture was determined by gravimetric analysis after drying at 105 °C until constant weight, and then converted to % water filled pore space (WFPS) using soil bulk density values measured on two occasions during the experiment.

Additional soil samples were collected from each plot 24 to 35 times (depending on the site) for the determination of soil mineral nitrogen (SMN) as ammonium (NH_4_^+^-N) and nitrate (NO_3_^−^-N). Only 15 samples were collected per plot from Crichton, as sampling started after the second N fertiliser application. No samples were taken from the AN80 and AN240 treatments, except from Hillsborough. Sample collection always coincided with days of N_2_O measurements and were conducted once per week in the month following the fertiliser application, and then sampling was reduced to a one- to two-month frequency. On each measurement day, five random samples of soil (0–10 cm depth) were taken per plot to obtain a representative bulk sample. Soil samples were crumbled or passed through a 2 mm sieve to remove stones and roots, and then extracted with KCl and filtered prior to analyses for NH_4_^+^-N and NO_3_^−^-N by colorimetric analysis (Searle, 1984).

### 2.6 Data analysis

Data analysis was carried out using GENSTAT Version 14.1. All data information was analysed by site. A normality test was carried out for all variables for each site separately. Data were normally distributed and therefore no transformation was necessary for the statistical analysis. The increase of grass N offtake and yield with increasing fertiliser N rate was fitted by linear regression. Exponential models were also tested to evaluate their correspondence with the law of diminishing returns, but for all sites the response to N fertiliser was found to be linear. Cumulative N_2_O emissions were calculated from the areas under the curve, following the method described in Cardenas et al. (2010).

An analysis of variance was carried out for each site to evaluate the effect of the treatments. The variables analysed related to herbage were grass dry matter yield, N offtake and grass N composition. Regarding N_2_O emissions, the variables analysed were accumulated N_2_O emissions, N_2_O EFs (expressed as a % of total N applied), yield-scaled N_2_O emissions (emissions per unit DM output) and emissions per amount of N offtake. Also, comparisons between sites and of the interaction between site and treatments were conducted using a mixed model and the restricted maximum likelihood (REML) algorithm, where block was a random effect. Differences were considered significant for P values < 0.05.

**Fig. 2:**
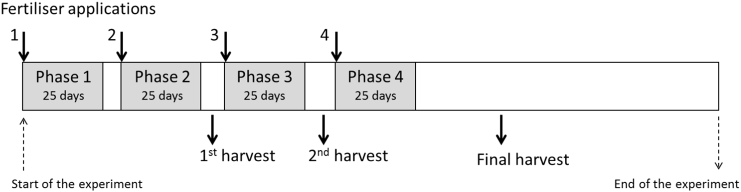
Time line for the experimental phases used for the analysis of the split application treatment. Duration of the phases have been normalised according to the shortest period between 2 fertiliser applications.

In order to explore the relationship between N_2_O emissions and N use by the crop in more detail, i.e. after each N fertiliser application, four phases were established comprising the first 25 days after each application (Fig. 2). Phase length was set to an equal number of days in order to normalise emissions, since the intervals between fertiliser applications were not identical at each site. For each phase, NUE, cumulative N_2_O emission and the N_2_O EF were calculated.

Finally, we used the annual averaged data to calculate correlations among the main variables analysed in this study. We also considered calculating correlations using specific data at the application times but no relevant results were found and therefore they are not presented in this manuscript.

The data from all the experiments were archived in the Agriculture & Environmental Data Archive (AEDA) and are available at http://www.environmentdata.org/.

## 3 Results

### 3.1 Weather conditions compared to long-term records

Weather conditions during the experiments differed from long term (28 years, from 1986 to 2013) averages depending on the measurement year, as shown in Table 3. Detailed weather conditions during the experiments compared to the long-term records are shown in the Supplementary material.

**Table 3:** Average annual temperature (°C) and total annual rainfall (mm) during the experiment compared to the long term (30 years) average, maximum and minimum year records.

	Experiment		Long-term temperature (annual average)			Long-term rainfall (annual total)
	Average annual temperature	Total annual rainfall	Average	Minimum	Maximum	Cumulative
Crichton	10.2	1209	9.1	5.5	12.8	1140
Drayton	10.1	771	10.3	8.6	11.3	628
North Wyke	9.9	1170	10.0	8.7	10.9	1042
Hillsborough	9.8	953	9.0	5.7	12.4	908
Pwllpeiran	9.9	1915	10.0	8.0	10.4	1570

Considering annual values, the accumulated rainfall during the experiment was higher than in long-term records, being 6, 24, 13, 6 and 22% higher for Crichton, Drayton, North Wyke, Hillsborough and Pwllpeiran, respectively. For those experiments carried out in 2011–2012 the average temperature was about 1 °C higher than the long-term records (+1.1 °C for Crichton and +0.8 for Hillsborough). In contrast, for experiments in 2013–2014 the average temperature was between 0.1 and 0.2 °C lower.

Seasonal weather variations from long term records were also detected. For Crichton and Hillsborough, March and April were relatively dry and warm months compared to the long-term records. However, from May to August the rainfall was greater than the average long-term records at Crichton and lower at Hillsborough. For the experiments in 2013–2014 (i.e. Drayton, North Wyke and Pwllpeiran), spring temperatures tended to be cooler than the long term records. April and June were dry months in those three sites, but May was normal compared to the long term record. In contrast, July was particularly dry both at North Wyke and Pwllpeiran.

### 3.2 Initial soil properties

Soil textures were similar at all sites (between clay and clay loam) except Crichton which was sandy loam, although the clay content varied between 23 and 59%. Crichton had 13% clay content. Soil pH was relatively acidic (pH 5.5–6.5) at all sites except for Drayton that was alkaline (pH 7.6). Soil organic carbon was similar at all sites (3.5–4.7%) except for Hillsborough, which was much higher at 9%. Together with North Wyke, Hillsborough had the lowest bulk density (<0.75 g cm^−3^) compared to the other sites (0.86–1.12 g cm^−3^). Hillsborough also had the highest initial available N and Olsen P contents compared to the rest of the sites.

### 3.3 Yields and N offtake

Crop yields are shown in Table 4. At all sites, the lowest yield came from the controls (from 2.78 t DM ha^−1^ at Drayton to 5.46 t DM ha^−1^ at Hillsborough). Site had a significant effect on DM yield production, with Hillsborough being the highest for all treatments, and the interaction between site and treatment was also significant. Yield significantly increased with N application rate and the highest values were obtained in the AN400 treatment (from 9.46 t DM ha^−1^ at Drayton to 16.97 t DM ha^−1^ at Hillsborough). Analysis showed that over the N application range studied, the response of yield to AN was linear up until the 320 kg N ha^−1^ application rate. No significant differences were found between the AN320 and AN400 treatments for any site. Increased split applications, and the use of DCD with AN did not significantly change the yield at any site. The application of urea reduced DM yield at all sites compared to the same N rate of AN (i.e. 320 kg ha^−1^), but these differences were not significant. The application of DCD did not significantly change yield, compared to the same treatment without the nitrification inhibitor.

**Table 4:** Herbage production results: grass yield, N offtake and N content of herbage. Standard errors (s.e.d.) are also provided.

	Increase in AN rates						Change in strategy at 320 kg N ha^−1^ application				
	C	AN80	AN160	AN240	AN320	AN400	AN320_NI	U320	U320_NI	AN320_Sp	s.e.d.
Yield (t DM ha^−1^)											
Crichton	3.85^a^	8.18^b^	9.72^bc^	10.81^c^	11.03^c^	11.28^c^	10.91^c^	10.74^c^	10.83^c^	11.16^c^	0.50
Drayton	2.78^a^	4.44^b^	6.30^c^	7.82^d^	9.32^e^	9.46^e^	9.28^e^	8.84^de^	7.95^d^	8.90^de^	0.33
North Wyke	4.22^a^	6.01^b^	7.59^b^	9.21^c^	10.54^cd^	10.89^d^	9.85^cd^	9.72^cd^	9.26^c^	10.29^cd^	0.45
Hillsborough	5.46^a^	9.65^b^	11.78^bc^	14.81^def^	15.11^def^	16.97^f^	15.34^def^	13.79^cd^	14.40^de^	16.70^ef^	0.68
Pwllpeiran	4.65^a^	7.25^b^	10.22^c^	11.37^cd^	12.00^d^	12.15^d^	12.38^d^	11.37^cd^	10.94^cd^	12.18^d^	0.45

N offtake (kg N ha^−1^ y^−1^)											
Crichton	49.7^a^	112.4^b^	162.0^c^	191.5^cd^	251.7^f^	286.8^g^	245.4^ef^	218.1^de^	224.1^ef^	285.3^g^	10.9
Drayton	54.6^a^	86.1^a^	132.6^b^	174.1^c^	197.5^cd^	241.2^e^	223.3^de^	205.1^cde^	173.6^c^	210.5^cde^	13.5
North Wyke	89.3^a^	129.7^a^	180.2^b^	247.3^c^	309.6^ef^	341.9^f^	275.3^cde^	262.0^cd^	244.1^c^	292.9^de^	14.7
Hillsborough	88.1^a^	158.3^b^	173.5^b^	282.5^c^	300.0^c^	362.7^d^	301.2^c^	256.7^c^	294.5^c^	304.8^cd^	20.2
Pwllpeiran	68.0^a^	115.4^b^	171.5^c^	252.9^ef^	274.1^f^	336.9^g^	269.5^f^	236.9^de^	217.4^d^	268.9^f^	7.34

N content of herbage (kg N kg^−1^ t DM)											
Crichton	13.1^a^	13.8^a^	16.7^b^	17.7^bc^	22.8^de^	25.4^ef^	22.5^d^	20.3^cd^	20.7^d^	25.6^f^	0.89
Drayton	19.7^a^	19.2^a^	21.0^ab^	22.2^abc^	21.1^abc^	25.5^d^	24.0^cd^	23.2^bcd^	21.9^abc^	23.6^bcd^	1.03
North Wyke	21.2^a^	21.5^a^	23.7^b^	26.8^cd^	29.4^e^	31.4^f^	27.9^de^	27.0^cd^	26.3^c^	28.4^de^	0.56
Hillsborough	16.0^ab^	16.0^ab^	14.7^a^	19.1^abc^	20.0^bc^	21.5^c^	19.7^bc^	18.6^abc^	20.5^bc^	18.3^abc^	1.59
Pwllpeiran	14.6^a^	15.9^ab^	16.8^b^	22.3^d^	22.9^d^	27.8^e^	21.9^cd^	20.8^cd^	19.9^c^	22.1^d^	0.74

Values with different letters (a to f) within a row indicate significant differences between treatments (P < 0.05).

Similar to DM yield, the total N offtake by the grass increased linearly with N fertiliser rate (see NUE results below and Table 4) between 0 and 400 kg N of AN application at rates of 0.58, 0.47, 0.68, 0.75, 0.68 kg N offtake per additional kg of N applied (R^2^ = 0.99 for all except Hillsborough with R^2^ = 0.98) for Crichton, Drayton, North Wyke, Hillsborough and Pwllpeiran respectively (resulting average is 0.63 kg N offtake per additional kg of N applied). When no N was applied, N offtake by the grass ranged between 50 and 89 kg N ha^−1^ (at Crichton and North Wyke, respectively), whereas for the AN400 treatment the offtake varied from 241 kg N ha^−1^ (Drayton) to 363 kg N ha^−1^ (Hillsborough). At the 320 kg N ha^−1^ rate, adding DCD, applying N in 6 splits or replacing AN by U (with or without DCD) caused a change in yield of <10% and the effect on N offtake was variable. Split application increased N offtake at Crichton, but not at the other sites. Urea application decreased N offtake compared to AN at Crichton, North Wyke and Pwllpeiran, but not at Drayton and Hillsborough. However, using DCD did not reduce N offtake with either AN or U fertilisers.

Site had a significant effect on the N content of grass, and the interaction between site and treatment was also significant (Table 4). The N content of grass increased with the N application rate (Table 4). When no N was applied, grass N content was lowest and ranged between 13.1 and 21.2 kg N t^−1^ DM at Crichton and North Wyke, respectively. In contrast, the highest grass N content was found when applying 400 kg N ha^−1^ year^−1^, and ranged from 25.4 to 31.4 kg N t^−1^ DM, at Crichton and North Wyke, respectively. In all treatments, the highest N content was found at North Wyke. However, at Hillsborough no statistical differences were detected between any of the treatments and at Drayton no statistical difference was detected between any treatment equivalent to 160 or more kg N ha^−1^. At the remaining sites, the grass N content at harvest was greater when using AN at a rate of 320 kg N ha^−1^ or more. Using urea, with or without DCD, did not significantly affect N content when compared to the equivalent N rate of AN.

**Fig. 3:**
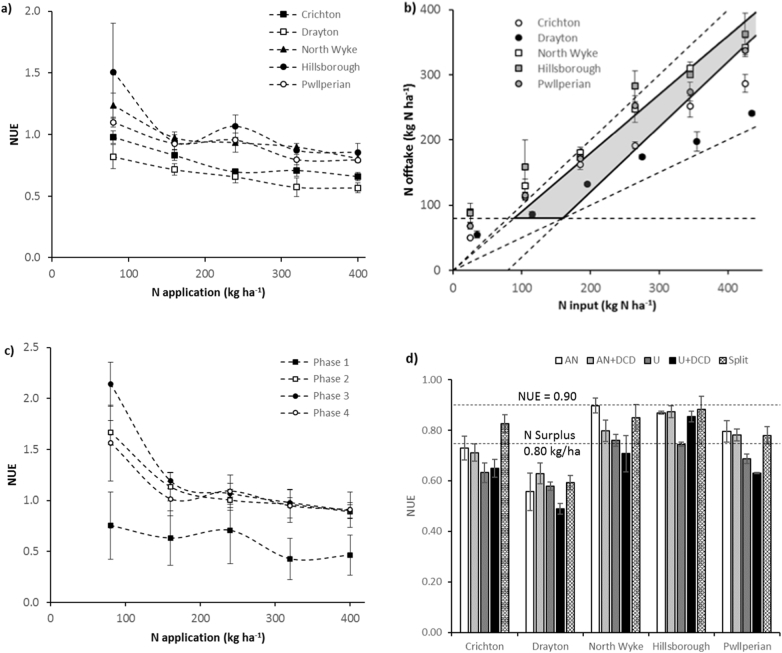
(a) Nitrogen use efficiency (NUE, kg N output/kg N input) as affected by nitrogen application rate at each site for the ammonium nitrate (AN) treatments; (b) the evaluation of NUE as affected by the N application rate, where N offtake is represented against N input (fertiliser N + atmospheric N deposition); the 90% and 50% NUE lines are represented, as well as the additional conditions of minimum offtake (80 kg N ha^−1^) and maximum surplus (80 kg N ha^−1^), also the shaded area represents the desirable range for NUE, N output and N surplus; (c) average NUE for all the sites for the AN treatments (excluding the changes in strategy) between successive fertilisation dates (phases 1, 2 and 3) and between the final fertiliser application and the final harvest (phase 4); and (d) effect of the application strategy for AN and urea at a rate of 320 kg N ha^−1^ on NUE in which the 90% NUE and 80 kg N ha^−1^ surplus (NUE = 0.75) thresholds are indicated as upper and lower dash lines, respectively.

The NUE results as affected by N application rate are represented in Fig. 3.a. NUE varied considerably between sites, but in all of them it decreased as N application rate increased. The lowest NUE values were found at Drayton (decreasing from 1.08 in the control to 0.60 in the AN400 treatment), whereas the highest NUE was found at Hillsborough (from 1.98 in the control to 0.91 in the AN400 treatment). These data are shown in Fig. 3.b as a two-dimensional input-output diagram, where the three main targets reported by the EU Nitrogen Expert Panel (2015) are presented: maximum NUE (90%), maximum N surplus (80 kg N ha^−1^) and minimum N offtake (80 kg N ha^−1^). At the AN rate of 320 kg N ha^−1^, NUE was within or near the recommended targets. This site fulfilled these targets only at the 80 and 160 kg N ha^−1^ application rate. When considering NUE for the phases after each fertiliser application separately (Fig. 3.c), the NUE was lowest during the period following the first application of fertiliser N, but it was higher for the periods following the rest of the fertiliser applications, which had similar NUE values.

The NUEs of fertiliser mitigation strategies at an application rate of 320 kg N ha^−1^ are also shown in Fig. 3.d. Strategies using AN (either in 4 or 6 splits, or adding DCD) had a higher NUE (P < 0.01) than those based on urea (either with or without DCD). The addition of a nitrification inhibitor, however, did not affect NUE. Site had a significant effect on NUE, but no interaction between site and fertiliser strategy was detected. For AN and AN + DCD, the highest NUE values were from North Wyke, Hillsborough and Pwllpeiran, and for the AN320 Split treatment they were high in all treatments except at Drayton.

### 3.4 Daily emissions of nitrous oxide

**Fig. 4:**
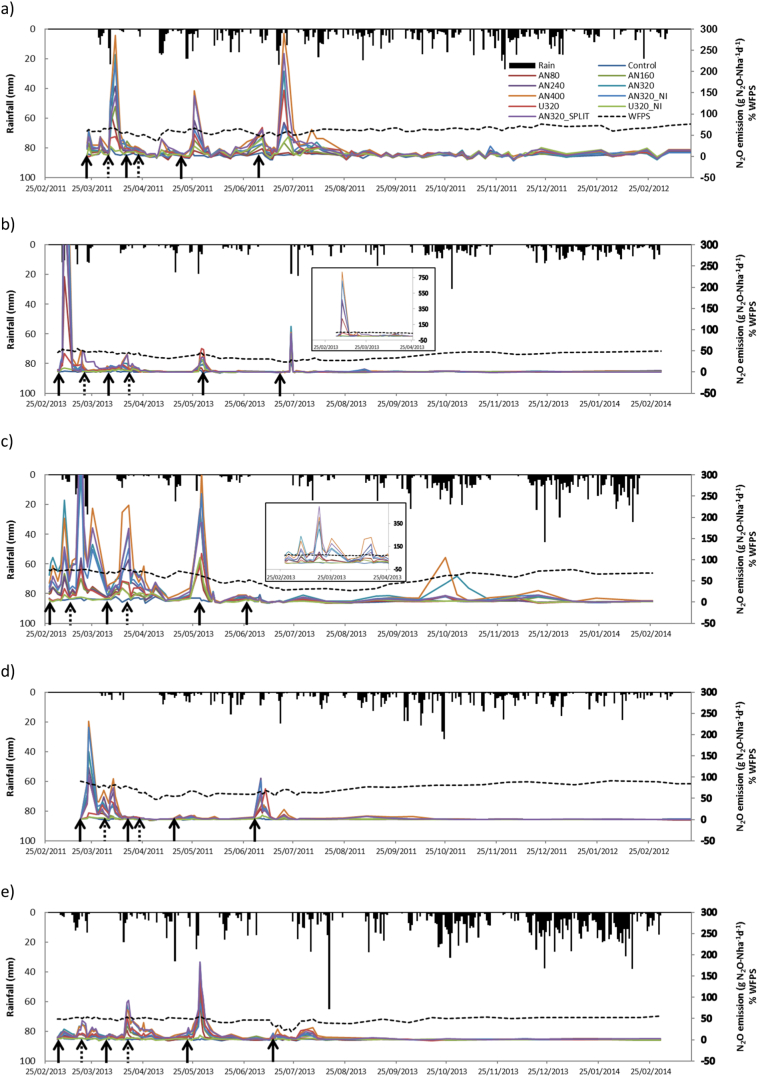
Evolution of daily N_2_O emissions, rainfall and WFPS at the measurement sites. a) Crichton, b) Drayton, c) North Wyke, d) Hillsborough, e) Pwllpeiran. Inserts in b) and c) show detail of the larger peaks. The continuous arrows correspond to fertiliser application; the dashed arrows correspond to the extra fertiliser additions in the split application treatments.

Daily N_2_O emissions per site and treatment are presented in Fig. 4. Nitrous oxide emissions increased after fertiliser application at all sites, but the response pattern was not consistent for all fertiliser application splits. The largest emission occurred after the first application at all sites except Crichton and Pwllpeiran, where a significant peak was also detected after the third application. The highest emission peaks were found from the AN400 treatment. In contrast, the smallest emissions were found from the control treatments. Emissions of N_2_O from the treatment with increased split applications generally showed a similar response to the corresponding treatment with conventional splits except in the first peak in all sites (excluding Pwllpeiran) which was higher for the conventional split treatment (however, this would not have been affected by the increase in split applications). At all sites, emissions were lower from the U treatment than from the corresponding AN treatment. The inclusion of an NI caused a reduction in emissions at all sites, both when applied with AN and with U, but it was only significant for Pwllpeiran. A more detailed analysis of daily N_2_O emissions per site follows (Fig. 4).

*Crichton*: Daily emissions were large, reaching about 300 g N_2_O-N ha^−1^ d^−1^ after the first and fourth fertiliser applications for the AN400 treatment. A third peak was observed after the third fertiliser application. The three observed emission peaks were produced after rain events. The soil moisture yearly average was 60% WFPS, with a range from 36 to 77%.

*Drayton*: A single main N_2_O peak was measured shortly after the first fertiliser application, also coinciding with a rainfall event. This peak was the largest measured at any of the sites (above 800 g N_2_O-N ha^−1^ d^−1^ for treatment AN400). Other smaller peaks (<100 g N_2_O-N ha^−1^ d^−1^) were observed after the third and fourth fertiliser applications. On average, soil moisture content was the lowest among the sites, and was on average 40% WFPS, ranging from 23 to 55%.

*North Wyke*: A main N_2_O peak (over 500 N_2_O-N ha^−1^ d^−1^ for the AN400 treatment) was observed 3 weeks after the first application. Another four significant peaks (above 200 g N_2_O-N ha^−1^ d^−1^) were detected during the first two months of the experiment. Additionally, a peak was measured in October from the two highest N rates (AN320 and AN400). Soil moisture was on average 61% WFPS and ranged between 27 and 80%.

*Hillsborough*: Most N_2_O emissions were produced after the first and fourth fertiliser applications. The main peak, produced shortly after the first application, was associated with high soil moisture (about 90% WFPS), despite the fact that no rainfall events occurred at this time. Very low emissions were measured after the second and third fertiliser applications. Soil moisture was on average 69% WFPS, and ranged from 47 to 91%.

*Pwllpeiran*: At this site, the largest N_2_O emission (182 g N_2_O-N ha^−1^ d^−1^ for treatment AN400) was found after the third application, coinciding with a rainfall event of 41 mm over the previous two days. Emissions were relatively low during the rest of the experiment. Soil moisture ranged between 20 and 56% WFPS and was on average 47%.

### 3.5 Cumulative emissions and EFs for nitrous oxide

The cumulative N_2_O emissions for all sites and treatments are shown in Table 5. Site had a significant effect on cumulative emissions, and the interaction between site and treatment was also significant. Cumulative emissions from the control treatments were the smallest at all sites and ranged between 0.20 (±0.01) and 1.34 (±0.17) kg N_2_O-N ha^−1^ y^−1^ at Hillsborough and Crichton, respectively. At all sites cumulative N_2_O emissions increased significantly with increasing AN fertiliser rate and the effect of increasing N fertiliser rate on annual N_2_O emissions showed linear responses for 3 of the 5 sites (R^2^ 0.97, 0.96 and 0.97 at Drayton, Hillsborough and Pwllpeiran, respectively), and exponential curves at the remaining 2 sites (R^2^ 0.99 and 0.95 at Crichton and North Wyke, respectively). Emissions from the AN400 treatment were between 6 and 16 times greater than from the control at Crichton and Drayton respectively, and generated the largest emissions across all sites. At the N rate of 320 kg N ha^−1^, the response to the various fertiliser strategies was different between sites.

**Table 5:** Cumulative N_2_O emissions during the experiment, emission factors, and yield-scaled emissions expressed per DM production and N offtake. Standard errors (s.e.d.) are also provided.

	Increase in AN rates						Change in strategy at 320 kg N ha^−1^ application				
	C	AN80	AN160	AN240	AN320	AN400	AN320_NI	U320	U320_NI	AN320_Sp	s.e.d.
Annual accumulated emission (kg N_2_O-N ha^−1^ y^−1^)											
Crichton	1.34^a^	2.18^ab^	3.15^ab^	4.28^bcd^	5.63^cd^	8.31^e^	4.53^bcd^	4.24^bcd^	3.26^abc^	6.50^de^	0.66
Drayton	0.23^a^	1.17^abcd^	1.89^bcde^	2.00^cde^	3.02^ef^	3.65^f^	2.97^ef^	1.00^abc^	0.49^ab^	2.52^def^	0.41
North Wyke	1.07^a^	2.70^ab^	3.14^ab^	4.33^abc^	10.22^de^	12.54^e^	7.13^bcd^	3.07^ab^	2.02^a^	8.97^cde^	1.41
Hillsborough	0.20^a^	0.74^ab^	1.14^abc^	1.74^bc^	1.62^bc^	2.81^d^	1.89^cd^	1.18^abc^	0.31^a^	1.61^bc^	0.28
Pwllpeiran	0.38^a^	0.95^abc^	1.37^bcd^	1.77^cd^	2.86^ef^	3.16^f^	1.56^cd^	2.06^de^	0.46^ab^	3.08^f^	0.26

Emission factor (% of N applied lost as N_2_O)											
Crichton	–	^yz^1.06^abc^	^z^1.14^abc^	^yz^1.23^abc^	^y^1.34^abc^	^y^1.74^c^	^y^1.00^abc^	^z^0.91^ab^	^z^0.60^a^	^y^1.61^bc^	0.23
Drayton	–	^yz^1.18^c^	^z^1.04^c^	^yz^0.74^bc^	^y^0.87^c^	^xy^0.86^c^	^xy^0.86^c^	^x^0.24^ab^	^y^0.08^a^	^x^0.71^bc^	0.15
North Wyke	–	^z^2.04^bcd^	^z^1.29^abc^	^z^1.36^abc^	^z^2.86^d^	^z^2.87^d^	^z^1.89^bcd^	^yz^0.63^ab^	^y^0.30^a^	^z^2.47^cd^	0.40
Hillsborough	–	^y^0.67^b^	^y^0.59^ab^	^yz^0.64^b^	^y^0.44^ab^	^x^0.65^b^	^wx^0.53^ab^	^xy^0.30^ab^	^y^0.03^a^	^x^0.44^ab^	0.17
Pwllpeiran	–	^y^0.71^bc^	^y^0.62^bc^	^y^0.58^bc^	^y^0.77^bc^	^xy^0.69^bc^	^w^0.37^ab^	^xy^0.52^bc^	^y^0.03^a^	^x^0.84^c^	0.12

Yield scaled emission (kg N_2_O-N kg^−1^ t dry matter)											
Crichton	0.35^ab^	0.27^a^	0.33^a^	0.40^ab^	0.52^bc^	0.74^d^	0.42^abc^	0.52^ab^	0.30^a^	0.58^cd^	0.06
Drayton	0.09^a^	0.27^b^	0.30^bc^	0.26^b^	0.32^bc^	0.39^c^	0.32^bc^	0.11^a^	0.06^a^	0.28^b^	0.04
North Wyke	0.26^a^	0.46^ab^	0.41^ab^	0.49^abc^	0.98^de^	1.17^e^	0.73^bcd^	0.32^a^	0.23^a^	0.88^cde^	0.14
Hillsborough	0.04^ab^	0.07^bc^	0.10^cd^	0.12^de^	0.11^cd^	0.16^e^	0.12^de^	0.08^cd^	0.02^a^	0.10^cd^	0.02
Pwllpeiran	0.08^ab^	0.13^bc^	0.14^cd^	0.16^cd^	0.24^e^	0.26^e^	0.13^bc^	0.18^d^	0.04^a^	0.25^e^	0.02

Emissions per kg N offtake (g N_2_O-N kg^−1^ N offtake)											
Crichton	26.96^b^	19.36^ab^	19.57^ab^	22.59^ab^	22.60^ab^	29.20^b^	18.61^ab^	19.76^ab^	14.71^a^	22.86^ab^	3.34
Drayton	4.12^ab^	14.05^cd^	14.50^d^	11.93^abcd^	15.36^d^	15.35^d^	13.33^bcd^	4.91^abc^	2.87^a^	11.97^abcd^	2.58
North Wyke	11.92^a^	21.64^abc^	17.48^ab^	18.25^abc^	33.39^bc^	37.0^c^	26.21^abc^	11.78^a^	8.79^a^	31.23^bc^	5.37
Hillsborough	2.36^ab^	4.90^abc^	6.75^bc^	6.07^bc^	5.40^abc^	7.9^c^	6.27^bc^	4.59^abc^	1.05^a^	5.40^abc^	1.38
Pwllpeiran	5.75^ab^	8.35^bcd^	8.01^bcd^	7.00^bc^	10.38^cd^	9.38^bcd^	5.74^ab^	8.71^bcd^	2.14^a^	11.52^d^	1.25

Values with different letters (a to f) within a row indicate significant differences between treatments (P < 0.05). For emission factors, values with different letters (w to z) within a column indicate significant differences between sites (P < 0.05).

Regarding cumulative emissions over the 25-day phases after each N fertiliser application, it was observed (see Supplementary material) that most emissions occurred after the first application, except for Pwllpeiran, where they occurred after the third application. For the AN320 treatment, a high proportion of the annual N_2_O emissions from Drayton and Hillsborough were measured during the 25 days following the first fertiliser application (76% and 58%, respectively), despite the fact that this accounted for only 7% of the measurement period. Emissions as a proportion of the annual emissions were found to be lower for the rest of the sites, ranging between 17% for Pwllpeiran and 31% for North Wyke. Cumulative emissions over the four successive 25 day periods after all four applications accounted for 56% (North Wyke) to 93% (Drayton) of the annual totals.

The resulting N_2_O EFs (expressed as a % of total N applied) are shown in Table 5. Increasing fertiliser rates of AN increased the EF only at Crichton and North Wyke; differences were only significant for North Wyke from treatments AN320 and AN400 compared to AN160 and AN240. Generally, North Wyke had the largest EF and Hillsborough the lowest for each N rate, but a wide variation of EFs was detected between sites. These were greater from North Wyke and Crichton, reaching 2.87% and 1.74% respectively, for the AN400 treatment. For the same treatment, the smallest EF was from Hillsborough, at 0.65%. Averages and standard errors of the EFs for all the applied rates had % values of 1.3 (0.12), 0.94 (0.08), 1.56 (0.45), 0.60 (0.04), 0.67(0.03) for Crichton, Drayton, North Wyke, Hillsborough and Pwllpeiran, respectively.

The yield-scaled emissions and the emissions per kg N offtake (N_2_O-N emitted per kg of N offtake by the grass) increased with N rate, but differences were only significant at the largest N rates for North Wyke and Hillsborough (Table 5).

### 3.6 Soil measurements

**Fig. 5:**
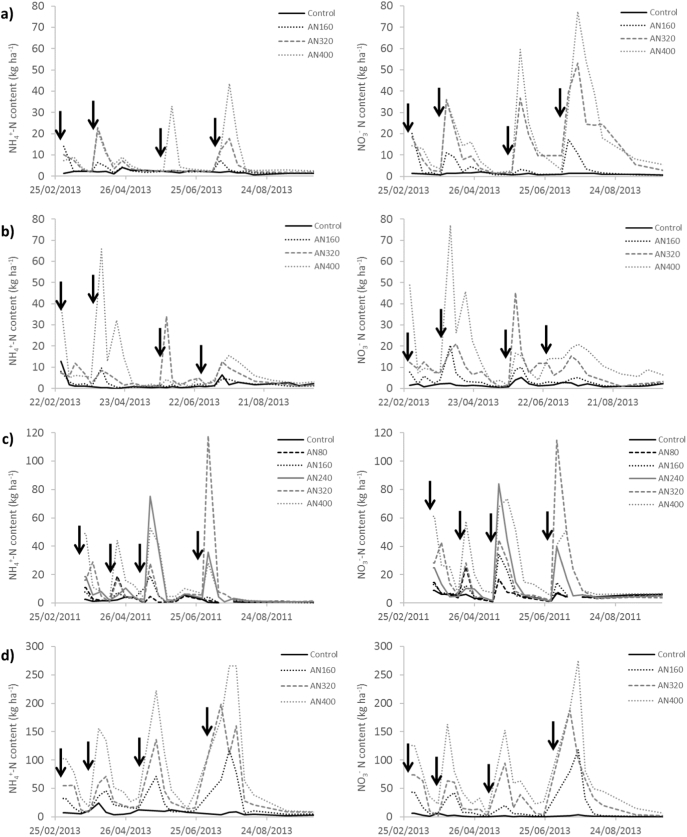
Soil mineral nitrogen content (NH_4_^+^ and NO_3_^−^) for the AN treatments at increasing rates from 0 to 400 N ha^−1^. Note that scales are different between sites. Data for Crichton is not presented because of missing data during the first two months of the experiment. a) Drayton, b) North Wyke, c) Hillsborough, d) Pwllpeiran.

**Fig. 6:**
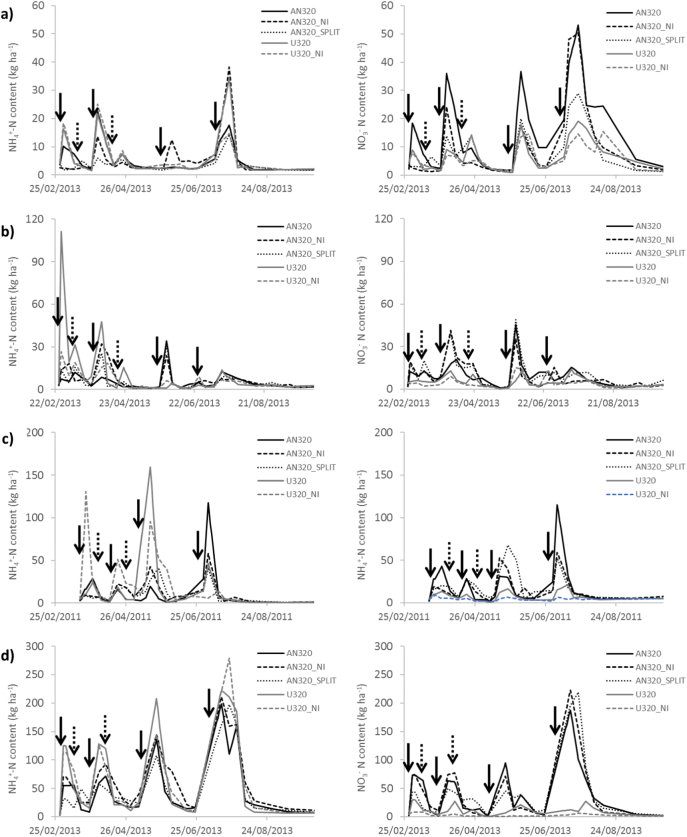
Time series of the soil mineral N concentrations (NH_4_^+^ and NO_3_^−^) for the treatments applied at 320 kg N ha^−1^: AN320, AN320_NI, AN320_Split, U320 and U320 + NI. Note that scales are different among sites. Data for Crichton is not presented because of missing data during the first two months of the experiment. a) Drayton, b) North Wyke, c) Hillsborough, d) Pwllpeiran.

Mineral nitrogen in the soil top 10 cm increased after each N fertiliser application, but decreased to relatively low values before the following application (Fig. 5, Fig. 6). Generally, both NH_4_^+^-N and NO_3_^−^-N reached similar values, but the greatest NH_4_^+^-N and NO_3_^−^-N contents were not always measured from the highest fertiliser N rate treatment. Values from Hillsborough and Pwllpeiran were greater than from Drayton and North Wyke. Table 6 shows the average soil NH_4_^+^-N and NO_3_^−^-N for each treatment and site at the end of the experiments. With few exceptions, there was an increase in soil N in all treatments compared to the initial soil values (Table 1), and compared to the corresponding control (Table 6). In all treatments, soil NH_4_^+^-N and NO_3_^−^-N values were greater than the control at the end of the experiments. In North Wyke and Pwllpeiran there was also an increase in the control compared to the initial soil NH_4_^+^-N. For NO_3_^−^-N, values were similar between the initial soil content and final content in the control. The value in the control in Pwllpeiran decreased relative to the initial value, whereas it increased in Hillsborough.

**Table 6:** Average soil ammonium (NH_4_^+^-N), nitrate (NO_3_^−^-N) and total mineral nitrogen (ammonium + nitrate) content per treatment and site at the end of the experiments.

	Increase in AN rates						Change in strategy at 320 kg N ha^−1^ application				
	C	AN80⁎	AN160	AN240⁎	AN320	AN400	AN320_NI	U320	U320_NI	AN320_Sp	s.e.d. treatment
NH_4_^+^-N (kg ha^−1^)											
Crichton⁎⁎	4.85^aBC^		6.08^abB^		10.63^abcB^	16.78^cB^	10.52^abcB^	10.90^abcA^	13.80^bcB^	11.12^abcB^	2.407
Drayton	2.00^aAB^		2.63^aAB^		3.71^abA^	5.43^bA^	4.07^abA^	4.05^abA^	3.98^abA^	2.87^abA^	0.733
North Wyke	1.92^aAB^		1.93^aA^		3.70^abA^	6.28^bA^	4.67^abAB^	5.93^bA^	3.88^abA^	4.00^abA^	0.961
Hillsborough	1.39^aA^	1.89^ab^	2.07^abA^	5.33^abc^	5.39^abcAB^	6.39^abcA^	5.45^abcAB^	8.01^bcA^	8.72^cAB^	4.80^abcA^	1.790
Pwllpeiran	6.17^aC^		16.92^aC^		31.31^bC^	48.76^cC^	40.08^bcC^	38.95^bcB^	40.02^bcC^	30.13^bC^	3.360
s.e.d. sites	0.91		1.15		1.74	2.53	1.79	3.42	2.19	1.04	

NO_3_^−^-N (kg ha^−1^)											
Crichton⁎⁎	9.10^aC^		15.75^abC^		28.94^bB^	28.67^b^	19.20^abB^	25.25^bB^	18.50^abB^	23.43^abB^	4.042
Drayton	1.07^aA^		2.37^abA^		9.41^cdA^	12.05^d^	6.16^bcA^	4.63^abA^	4.36^abA^	5.18^bA^	1.028
North Wyke	2.49^aA^		4.04^abAB^		7.03^abcA^	12.55^c^	7.61^abcA^	5.52^abA^	4.48^abA^	8.67^bcA^	1.523
Hillsborough	4.97^aB^	5.23^a^	5.98^aAB^	8.40^ab^	9.81^abA^	14.18^b^	10.37^abA^	5.45^aA^	4.52^aA^	11.08^abA^	2.024
Pwllpeiran	1.09^aA^		10.66^abBC^		23.30^cAB^	39.77^d^	24.12^cB^	6.25^abA^	2.20^aA^	24.22^cB^	3.257
s.e.d. sites	0.65		2.05		5.21	3.58	2.46	2.55	1.60	2.19	

Mineral nitrogen (kg ha^−1^)											
Crichton⁎⁎	13.95^aB^		21.83^ab^		39.57^bcB^	45.44^cB^	29.72^abcB^	36.15^bcB^	32.30^abcB^	34.55^abcB^	5.90
Drayton	3.07^aA^		5.00^ab^		13.12^cdA^	17.48^dA^	10.23^bcA^	8.69^bcA^	8.34^abcA^	8.05^abcA^	1.469
North Wyke	4.41^aA^		5.97^ab^		10.73^abA^	18.83^cA^	12.28^abcA^	11.45^abAc^	8.36^abA^	12.67^bcA^	2.153
Hillsborough	6.36^aA^	7.12^a^	8.05^a^	13.72^ab^	15.20^abA^	20.57^bA^	15.81^abAB^	13.46^abA^	13.24^abA^	15.87^abA^	3.325
Pwllpeiran	7.26^aA^		27.58^ab^		54.61^cdB^	88.53^eC^	64.20^dC^	45.21^bcdB^	42.22^bcB^	54.35^cdC^	5.66
s.e.d. sites	1.38		3.07		6.80	5.14	4.18	5.31	3.29	2.94	

⁎ For the AN80 and AN240 treatments, mineral nitrogen was only measured in Hillsborough. Small letters are the comparison of treatments for each site (P < 0.05), the capital letters are the comparison of sites for each treatment (P < 0.001).

⁎⁎ At Crichton measurements of soil mineral started two months after the start of the experiment.

Soil moisture as WFPS (data not shown) was lower (down to 40% WFPS) in the summer in the experiments at Crichton and Hillsborough (2011−2012) and increased later (up to 100% WFPS) in the autumn/winter period. At the other three sites (2013–2014), moisture was very low (20–30% WFPS) in the middle of the summer and higher in the spring and the autumn (up to 50% at Drayton and Pwllpeiran, and 80% in North Wyke).

### 3.7 Mitigation strategies

The most effective strategy for reducing the N_2_O EF was applying urea plus the nitrification inhibitor, DCD (85% reduction on average compared to the equivalent AN320 treatment), followed by using urea instead of AN (49% reduction) and adding DCD to AN (19% reduction). Increasing the number of N fertiliser split applications was not effective (1% reduction on average). A strong interaction between site and mitigation strategy (P < 0.001) indicated that site conditions influenced the effectiveness of the mitigation strategies.

#### 3.7.1 Application of urea, and urea with NI

Replacing AN with urea reduced yield-scale emissions at all sites (significant differences only at Drayton and North Wyke). Adding DCD to urea additionally reduced emissions at all sites, although this further reduction was only significant at Pwllpeiran and emissions for this treatment were not significantly different from the control at any site. The combined effect of Urea + DCD reduced emissions significantly with respect to AN at Drayton, North Wyke and Pwllpeiran. Although not significant, this combination also reduced yield-scaled emissions at Crichton and Hillsborough, by 34% and 80%, respectively.

Using urea was particularly effective in reducing EFs at Drayton and North Wyke (72% and 78% reduction in the EF compared to using AN), but less so at Crichton, Hillsborough and Pwllpeiran (about 32% reduction on average). Combining urea with DCD was very effective at all sites (EF reduction of over 90%) except for Crichton (55% reduction in EF). Adding DCD to urea was an effective way to reduce annual emissions, but was only significant at Pwllpeiran and.

Urea applications had the smallest EFs at all sites, particularly when DCD was applied. Urea application significantly reduced the EF only at Drayton and North Wyke, when compared to the equivalent AN treatment, but Urea + DCD significantly reduced EFs at all sites except for Crichton.

#### 3.7.2 Application of NI with AN

Adding DCD to AN reduced the EF by 53% at Pwllpeiran, but increased it by 19% at Hillsborough. Applying DCD to AN reduced annual N_2_O emissions except at Hillsborough and only significantly at Pwllpeiran. Comparison of the EFs between sites showed that the largest EFs were from North Wyke for the AN320_NI and AN320_SP treatments, and largest at Crichton for the U320 and U320_NI treatments. Adding DCD to AN reduced yield-scaled emissions at all sites except Drayton and Hillsborough, although the reduction was only significant at Pwllpeiran.

#### 3.7.3 Increasing the number of fertiliser applications

Increasing the number of split N fertiliser applications had no significant effect N_2_O emissions at any site. In addition, no effect of splitting AN application on yield scaled emissions could be detected.

## 4 Discussion

### 4.1 Effect of weather and initial soil conditions on emissions

Experiments were carried out in different years: 2011–2012 at Crichton and Hillsborough, and 2013–2014 at Drayton, North Wyke and Pwllpeiran. Therefore, comparison between sites must take into consideration that a combination of different weather conditions including rainfall and temperature and soil properties may explain differences. The data however, did not show correlations between total yearly emissions and temperature or rainfall during the experiments (15% for emissions vs temperature, −2% for emissions vs rainfall) unlike the similar study by Bell et al. (2015) on three sites on arable land who found a strong influence from rainfall (in their study all sites had experiments carried out in the same year). A study on arable crops in China also showed that log transformed N_2_O fluxes were correlated with soil temperature and WFPS (Li et al., 2017) whereas on another study, Fan et al. (2018) found emissions related to soil pH and nitrate contents. Although weather conditions are considered to be particularly relevant after fertiliser application times when most N_2_O emissions are produced (typically from March to July), it has been observed that the conditions prior to the application of fertiliser can be the driving factor in the resulting fluxes (Bergstermann et al., 2011). However, ten days before fertiliser was applied in all sites, rainfall was variable (data not shown) and cannot explain the differences in total emissions.

The clay contents of the soils did not seem to show a direct effect on emissions, as Drayton for example, with the highest clay values, gave low cumulative emissions. Previous studies have shown close links between texture and emissions, with larger emissions in fine-textured soils due to reduced aeration, but this link was also related to soil moisture (McTaggart et al., 2002). Similarly, SOC could not explain the emissions trend, as North Wyke for example had high emissions but relatively low SOC content and Hillsborough had the highest SOC (and highest initial N) and lowest cumulative emissions, unlike other studies (Harrison-Kirk et al., 2015) where high SOC seemed to result in higher fluxes. Detailed laboratory studies have shown that there is interaction between variables and these interactions are likely to vary for different soil types. We don't have enough information in our study to define these interactions and this would require laboratory studies that assess these effects in a multivariate framework under controlled conditions (García-Marco et al., 2014).

### 4.2 Nitrous oxide emission and emission factors

The rapid response of the N_2_O emissions to fertiliser addition at all our sites is unlike the findings of Bell et al. (2015), who observed a delay up until the last fertiliser application and attributed this to a limiting environmental factor. However, in their study they experienced unusually dry weather whereas in our case the climate during the experimental period was relatively wet in the spring and only in the 2013 experiments was dry weather experienced in the middle of the summer. The exponential response of total emissions to fertiliser rate at Crichton and North Wyke agrees with previous results at a site near the latter and at two other sites located in England and Wales that were grazed (Cardenas et al., 2010). Other studies have found similar responses, and out of 26 datasets only 4 showed linearity and 16 were of an exponential nature. This was explained by excessive N supply relative to plant and microbial demand (Kim and Kim, 2013).

The overall average annual EF for the five AN nitrogen application rates (from 80 to 400 kg N ha^−1^) of 1.12% total N applied, is slightly higher than the default Tier 1, IPCC value of 1% (IPCC, 2006). However, the wide range of EFs found for the five different sites, (average value EF for the five AN application rates), was shown to be highly variable, from about half (0.60% at Hillsborough) to just over double (2.08% at North Wyke) the IPCC default value.

The values of temperature and rainfall during the experiments at all the sites were similar to the long term values (only over 20% higher in rainfall for Pwllpeiran and Drayton), suggesting that the climate was representative of a typical year, and in consequence suggesting that the EFs obtained here can be used as good estimates for these regions in the UK.

### 4.3 Grass yields, N offtake and nitrogen use efficiency

The effect of site on yield could not be explained by the soil pH, but there was a correlation with clay (−37%) and SOC (40%) content. Soil factors (including N content), and management, seem to be affecting grassland production in interaction with weather conditions. Remarkably, the differences in yield and N offtake between sites for the control treatment were maintained for all fertiliser treatments. The smallest and largest yields corresponded in all treatments to Drayton and Hillsborough, respectively. Similarly, N_2_O emissions were greater at Crichton and North Wyke throughout the different application rates and fertiliser strategies. Conversely, Drayton, Hillsborough and Pwllpeiran had the smallest emissions from all treatments including the control. Both yield scale emissions expressed relative to the N content or dry matter content of the pasture, showed a similar trend of increase with increase in N rate application, showing that either can be used to determine the impact of fertilisation on product emissions.

The results of grass N offtake showed that not all N added was used by the plants, resulting in an average surplus of 0.32 kg N per additional kg N applied (R^2^ = 0.99). This means that the maximum surplus of 80 kg N ha^−1^ recommended by the EU Nitrogen Expert Panel (2015) was reached on average at an AN application rate of 320 kg N ha^−1^. N offtake was highly correlated with fertiliser rate and soil pH by 94 and 63%, respectively. Low correlations were found with clay and SOC content, at 12 and 15%, respectively. As with other variables, relevant differences were found between sites, indicating a strong site effect where weather and soil conditions are likely to be important.

The surplus N also increased linearly with N application, resulting in an increased risk of loss to the environment. When N_2_O emissions were expressed as a proportion of N surplus, we found that this value was much greater for North Wyke (31% for the AN320 treatment) than for the rest of the sites (from 2 to 6%). The surplus is a proxy for losses, so in the case of North Wyke a significant proportion of the surplus goes as N_2_O emissions, whereas in the other sites there might have been other major losses such as NH_3_ volatilisation, N leaching or N immobilisation (that were not measured). The correlation between N offtake and N_2_O emissions was high at 63%. However, no significant correlations among NUE, N excess and N_2_O were found.

The resulting NUE values from our study are higher than the “System N efficiency” values reported by Godinot et al. (2015): 50% for crops and between 10 and 30% for beef cattle. Their values include other parameters such as the N used for the production and transport of net inputs, as well as soil N variations which we don't have in our calculations.

The lowest NUE found in Phase 1, coincided with the largest emissions. This would suggest that early in the season the plant is less efficient in taking up nitrogen, causing higher losses.

The results from this study placed in the context of the EU targets for NUE showed that the values might have to be slightly adjusted for the UK (values in the NUE report are conceptual), as for low N application rates the N offtake is higher than the 90% target value for all sites. These values would apply to cut grasslands, but not necessarily to grazed grasslands. For high N application rates, values for two of the sites are outside the desirable area as shown in Fig. 3 (Crichton and Drayton) but still above the 50% NUE target.

### 4.4 Mitigation strategies

Although applications of urea and particularly urea with DCD generated the smallest N_2_O emissions, the NUE tended to be lower from these strategies than from the corresponding AN320 treatment, due to lower grass yields and N offtake. Consequently, the N surplus for the urea treatments applied at the 320 kg N ha^−1^ rate were comparable to that for the AN rate of 400 kg N ha^−1^. Losses of NH_3_ from N fertilisers are estimated to be 10% (IPCC, 2006) which for urea represents a higher fraction due to its larger NH_4_^+^ content compared to AN and CAN. Our results differ from the similar study on arable sites by Bell et al. (2015) as they found no effect of site and no difference if the NI was applied to Urea or AN. It is worth mentioning that other inhibitors such as NBPT, an urease inhibitor, have shown effective reduction in emissions (Forrestal et al., 2017), but we did not test this. Bell et al. (2015) also did not find a difference between Urea and AN, whereas we found a significant effect at two sites. The lower EFs from urea suggest that denitrification was the dominant source of emissions in all sites. Results from Lebender et al. (2014) differed from ours as they showed that EFs in arable soils from urea application were larger than calcium ammonium nitrate and this was attributed to a temporal increase in pH with urea application, causing nitrite to accumulate. For the split fertiliser application treatment, Bell et al. (2015) found an effect, unlike in our experiments. Our lower EFs from urea compared to AN when adding DCD, align with the lower EF found when applying urea vs AN and no DCD is applied. We assumed that most of the emissions were produced from denitrification which means that the effect of the inhibitor was of an indirect nature, via inhibiting nitrification, emissions were reduced during denitrification.

It seems from our results and those of Bell et al. (2015) that mitigation strategies have different effectiveness in arable than in grassland soils. For example, Roche et al. (2016) found a lower emission factor for CAN and no difference between urea and CAN. Urea seems a better choice for grassland in terms of emissions, but care needs to be taken when NUE is considered and N offtake needs to be optimised. The application of DCD on grassland seems to be worth considering, but not further split application of N fertiliser. Issues have been raised when using this nitrification inhibitor, as traces of DCD were found in milk when DCD was directly fed to animals (Welten et al., 2016), and grass leaves from grasslands to which DCD had been applied have been reported to contain traces of DCD (Pal et al., 2016).

The varied responses to the various fertiliser strategies at the different sites suggest that mitigation measures not only need to consider the land use and management but also must be tailored to location, taking into consideration soil type and climate.

### 4.5 Correlations between nitrogen parameters and other factors

A correlation matrix between averages of cumulative N_2_O emissions, EFs, grass yield, Nofftake, soil mineral N content, total N input, NUE and Nexcess showed that total N input was related to Nofftake and Nexcess; cumulative emissions and EF related to yield scaled emissions (all over 75% correlation coefficient) (Table 7). When the yearly rainfall and mean air temperature were also included in the matrix, rainfall was shown to correlate with soil mineral N content, whereas temperature showed no correlation with any of the parameters. Although it seems that precipitation around the time of fertiliser application plays an essential role, our results reported similar emissions at Hillsborough and Pwllpeiran, with very different rainfall patterns. It seems therefore that under practical conditions a variety of factors and their evolution throughout the growing session may explain the magnitude of the final N_2_O emission.

**Table 7:** Correlation matrix between averages of cumulative N_2_O emissions (N_2_O), grass yield dry matter (DM), N offtake, soil mineral nitrogen (SMN), N_2_O emission factor (EF), yield scaled emissions, N rate, Total input N including atmospheric deposition, nitrogen use efficiency (NUE) and N excess. Significant correlations were identified: P > 0.05 (*), P > 0.01 (**) and P < 0.001 (***), values with the latter significance in bold.

	N_2_O	Yield	N offtake	SMN	EF	Yield scaled emissions	N rate	Total input N	NUE	N excess
N_2_O	1.00									
Yield	0.14	1.00								
N offtake	0.56^⁎⁎^	**0.80**^⁎⁎⁎^	1.00							
SMN	0.10	0.24	0.45	1.00						
EF	**0.87**^⁎⁎⁎^	−0.21	0.21	−0.09	1.00					
Yield scaled emissions	**0.89**^⁎⁎⁎^	−0.22	0.19	−0.01	**0.93**^⁎⁎⁎^	1.00				
N rate	0.58^⁎⁎^	**0.64**^⁎⁎⁎^	**0.90**^⁎⁎⁎^	0.49^⁎^	0.18	0.27	1.00			
Total input N	0.57^⁎⁎^	**0.63**^⁎⁎⁎^	**0.89**^⁎⁎⁎^	0.48	0.18	0.26	**1.00**^⁎⁎⁎^	1.00		
NUE	−0.26	−0.04	−0.26	−0.24	0.01	−0.20	−0.61	−0.63	1.00	
N excess	0.36	0.13	0.38	0.30	0.06	0.27	**0.74**^⁎⁎⁎^	**0.76**^⁎⁎⁎^	−**0.91**^⁎⁎⁎^	1.00

N fertiliser application increased yield at all sites as expected, but the different response functions do not seem to be explained by soil properties, as for example the different soil textures; Pwllpeiran had a similar texture to Hillsborough and North Wyke, yet the response was similar to that at Crichton, which had a sandy loam texture. The higher yields at Hillsborough and Pwllpeiran may be explained by the initial SMN contents. The low yields at Drayton may be explained by the lower rainfall compared to all other sites as well as lower initial SMN. The rate of increase in herbage N with fertiliser rate on the other hand was greatest from the three clay loam soils, which also coincided with larger values for the zero N treatment, from 68 to 89.3 kg N ha^−1^, suggesting some priming for N offtake could have occurred, as the other two sites had less N in the control treatment. This could be explained by the N addition to soils causing priming effects due to stimulation of microbial mobilization of native N bound within pre-existing soil organic matter (Kim et al., 2013). The response curves also showed that there still seemed to be potential for further offtake, as a plateau had not been reached at the highest N rate, which was not the case for yield at Crichton, Hillsborough and Pwllpeiran. This suggests that for these three sites there is more potential for further N_2_O losses at high N application rates.

Yields were generally the largest and EFs the smallest at Hillsborough, possibly due to the high initial SOC (Table 1) promoting full denitrification. It is also likely that the lack of rainfall before fertiliser was applied caused the low emissions. Emission factors from CAN application have been reported to be higher than from urea and larger than the IPCC default (Harty et al., 2016), and also higher than from AN (Smith et al., 2012). The values reported by Harty et al. (2016) were 1.49% and 0.25% for CAN and urea, respectively, which corresponded to a N rate application of 200 kg N ha^−1^. The closest rate in our experiment of 240 kg N ha^−1^ gave an EF of 0.64% for AN, much lower than for CAN, the closest fertiliser form used in Harty et al. (2016).

The mitigation strategies tested resulted in more efficient N use when AN was applied, probably, in the case of DCD, due to immediate inhibition of nitrification of the NH_4_^+^-N, less effective in the urea treatment as the ratio inhibitor to NH_4_^+^ is lower. Generally, increasing the number of N fertiliser application splits resulted in a higher NUE than from other mitigation strategies, which shows the importance of matching grass demand and supply of N. Drayton was generally less efficient in NUE probably due to a combination of factors. The higher surplus and lower Nofftake values in all cases suggest N is easily lost in this soil/climate combination before the plant has a chance to take it up. The rainfall during the experiment year was slightly higher than the average long term rainfall and could have promoted N losses as N_2_, also motivated by the higher soil pH. In the previous study by Chadwick et al. (2018) where urine and dung were applied on 3 different times of the year, this site also gave in general the lowest N_2_O emissions compared to the other sites included in the study. It is also possible losses as NH_3_ occurred (stimulated by the high pH) but we did not measure these.

The effect of the tested mitigation strategies resulted in a decrease in the EF for DCD application, with AN larger than U, and both larger than reported values from a meta-analysis carried out by Gilsanz et al. (2016), where for a grassland on a clay-clay loam with an ammonium based fertiliser the EF was 0.39 ± 0.08%. For a grassland on a sandy loam, as in the case of Crichton, with an AN based fertiliser, the value reported was 0.01 ± 0.02%, much lower than our findings. Their study included data from Europe, Australia, China, New Zealand, and India (*pers. comm.*) and their results suggest that mitigation measures need to be site specific and cannot be applied with the hope that they will work; certainty of their effectiveness needs to be obtained. We suggest that these variations are included in models and practical tools are required so farmers can apply the right strategy for the right place at the right time.

## 5 Conclusions

The results from our study showed that for the UK sites studied between 2011 and 2014, increasing rates of application of N as ammonium nitrate (AN) increased yield, NUE and N_2_O emissions. N_2_O emissions were greater at Crichton and North Wyke whereas Drayton, Hillsborough and Pwllpeiran had the smallest emissions. The overall average annual EF for the five AN nitrogen application rates (from 80 to 400 kg N ha^−1^) of 1.12% total N applied, was slightly higher than the default Tier 1, IPCC value of 1%. The values were highly variable between sites: between 0.6 and 2.08%.

The NUE was lower from the treatments that included application of urea and urea with DCD, and they generated the smallest N_2_O emissions, when compared to the corresponding AN320 treatment. The maximum surplus of 80 kg N ha^−1^ recommended by the EU Nitrogen Expert Panel (2015) was reached on average at an AN application rate of 320 kg N ha^−1^. The results from our study placed in the context of the EU targets for NUE showed that the values might have to be slightly adjusted for the UK. Yield scaled emissions and emissions expressed as a fraction of the N content of the pasture, showed an increase with the increase of N rate application. The mitigation strategies tested showed great potential for increasing NUE with the use of DCD with AN, and increasing the number of N applications, demonstrating the need for further exploring better, more accepted inhibitors and ensuring that the supply of N matches the need of the pasture. Tools to mitigate emissions should be made available so farmers can apply the right strategy for the right place at the right time.

## Appendix A Supplementary data

Supplementary material Image 1
