# Supplementary material for: Nitrogen use efficiency and nitrous oxide emissions from five UK fertilised grasslands
Source: Sci Total Environ. 2019 Apr 15;661:696–710. doi: 10.1016/j.scitotenv.2019.01.082 (PMC6383039; doi:10.1016/j.scitotenv.2019.01.082)
Supplement: Supplementary file 1 — Supplementary material [file mmc1.docx]

**Supplementary material 1: Met. data during the experiment compared to the long term (30 yr) usual values, expressed as 20 and 80% percentiles.**

Experiments during year 2011: Normal temperatures, except for April and autumn (warmer). Variable rainfall along the year.


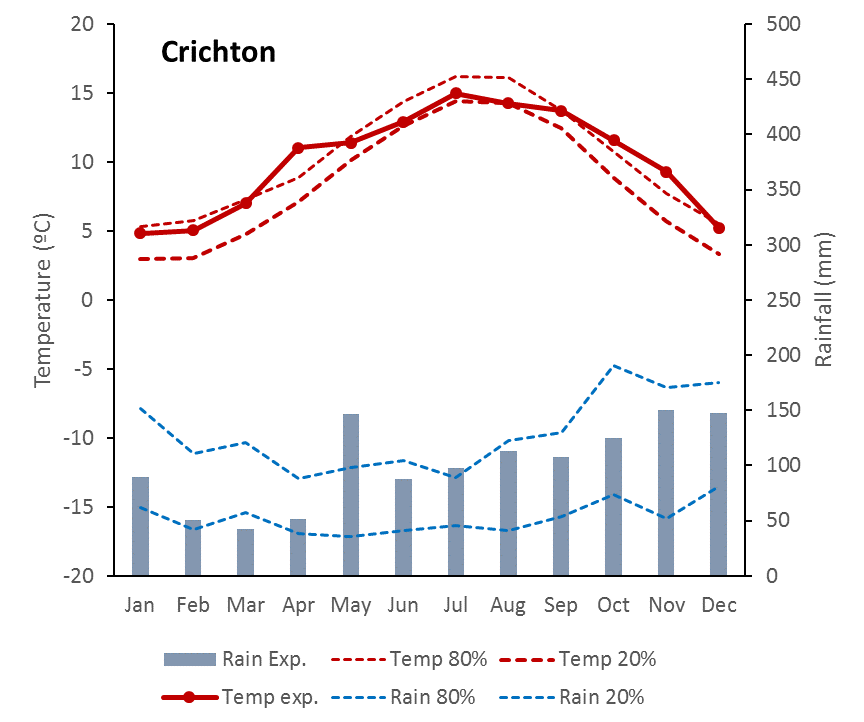

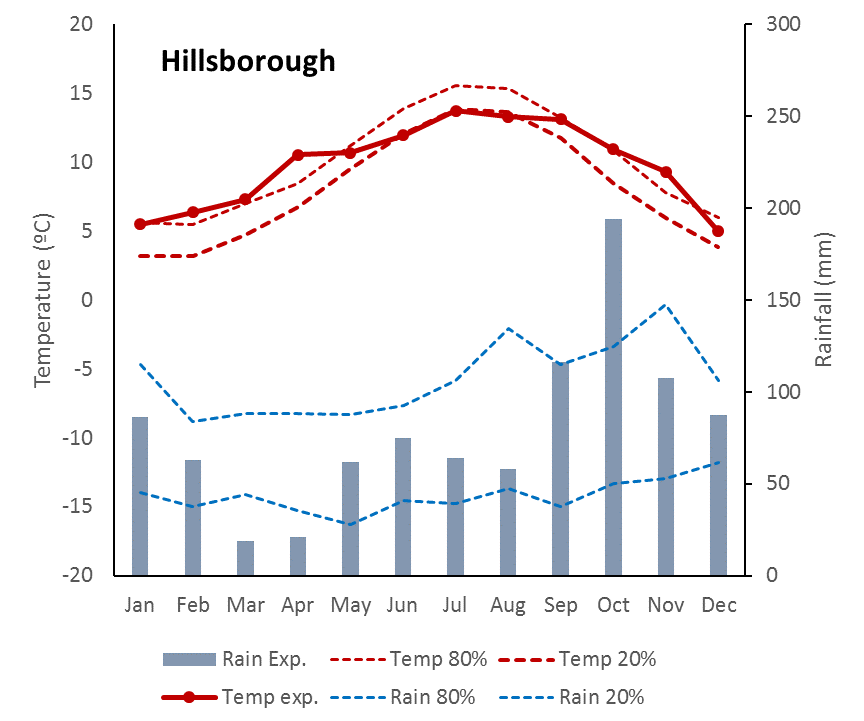


Experiments during year 2013: Temperatures mostly within the usual ranges. Variable rainfall patterns.


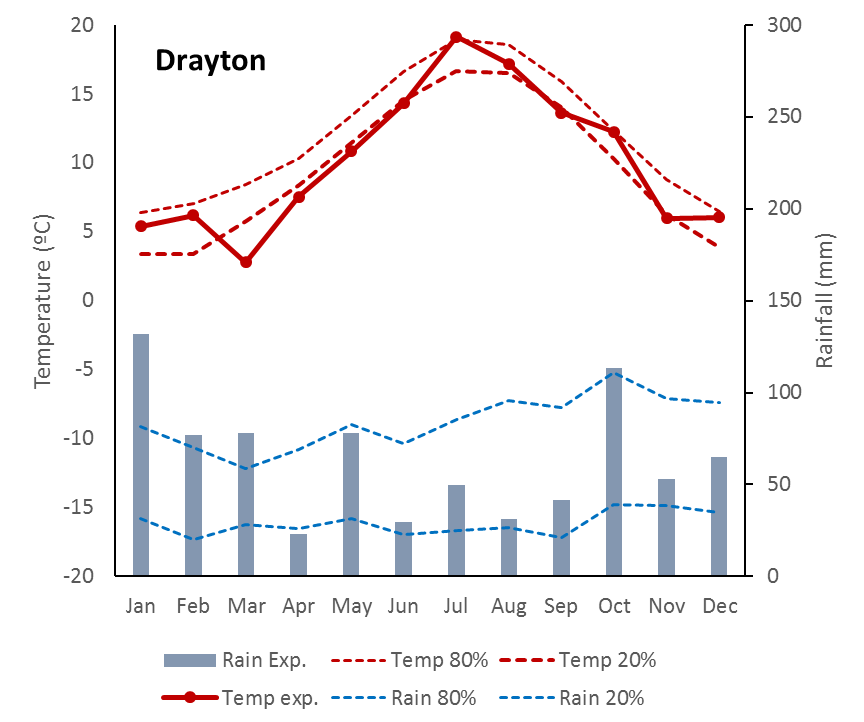

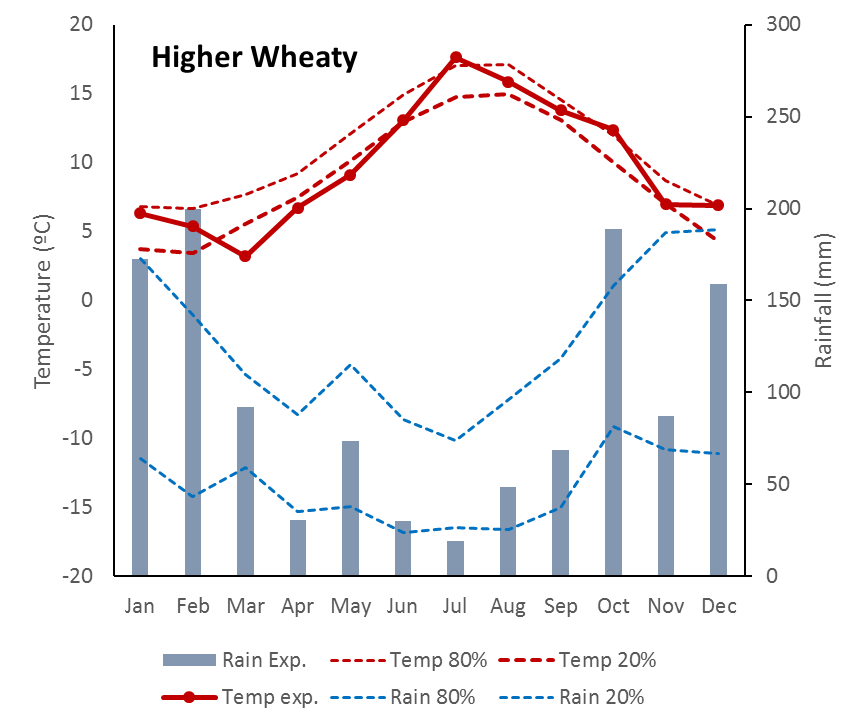


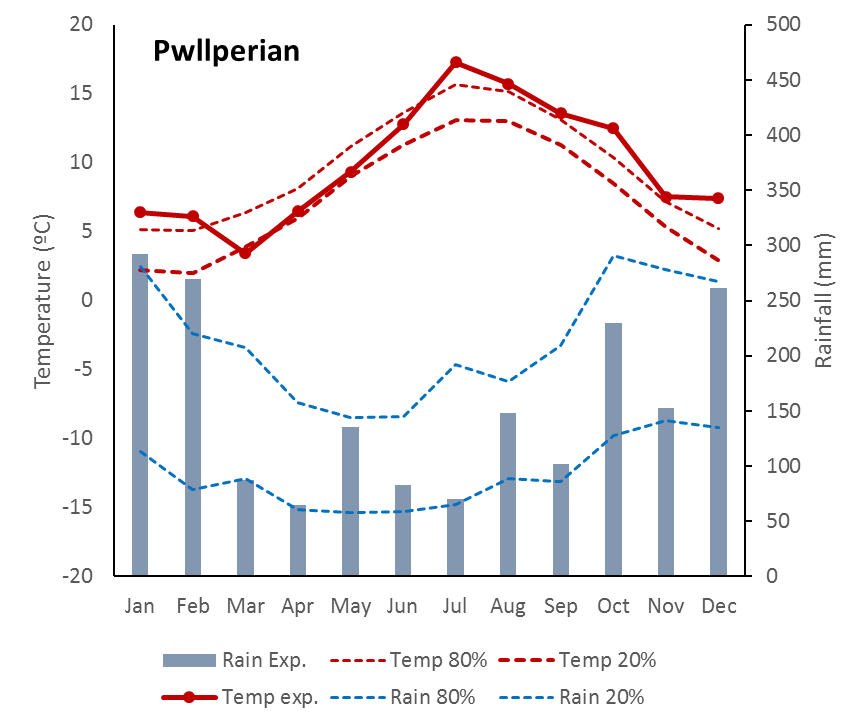


**Supplementary Material 2: N_2_O emissions per phase of fertiliser application.** Each phase (from 1 to 4) includes the 25 days following each fertiliser application. N_2_O emissions (g N_2_O /ha) accumulated during these periods are reported. Average ambient data is also included: Water filled pore space (WFPS, %), air temperature (Air T, ºC) and accumulated Rainfall (mm)

| **Phase** | **Site** | **N_2_O emissions** | | | | | | | | | | **Ambient** | | |
| --- | --- | --- | --- | --- | --- | --- | --- | --- | --- | --- | --- | --- | --- | --- |
|  |  | **Control** | **AN80** | **AN160** | **AN240** | **AN320** | **AN400** | **AN320_NI** | **U320** | **U320_NI** | **AN320_SP** | **WFPS** | **Air T** | **Rainfall** |
| **1** | Crichton | 188.5 | 672.1 | 868.9 | 1202.0 | 1291.6 | 1816.6 | 1426.6 | 500.1 | 249.7 | 1069.1 | 9,39 | 8,65 | 65,7 |
|  | Drayton | 26.0 | 743.9 | 1392.7 | 1376.0 | 2308.3 | 2832.2 | 2325.8 | 342.3 | 87.4 | 1811.6 | 2,75 | 3,48 | 67,6 |
|  | North Wyke | 152.5 | 854.2 | 793.9 | 936.5 | 3207.0 | 3178.4 | 2094.6 | 577.7 | 210.1 | 4199.9 | 3,87 | 4,93 | 92,2 |
|  | Hillsborough | 46.3 | 430.4 | 708.1 | 988.3 | 938.1 | 1663.4 | 1170.6 | 443.4 | 80.1 | 780.8 | 9,31 | 9,94 | 23,6 |
|  | Pwllperian | 62.6 | 230.1 | 188.5 | 206.1 | 491.0 | 413.6 | 212.3 | 199.7 | 68.2 | 511.2 | 3,35 | 3,84 | 68,5 |
| **2** | Crichton | 89.7 | 93.7 | 235.3 | 147.2 | 260.9 | 391.1 | 179.1 | 213.7 | 152.2 | 492.3 | 11,76 | 11,64 | 71,5 |
|  | Drayton | 32.0 | 133.7 | 174.7 | 156.8 | 246.1 | 281.1 | 220.9 | 189.2 | 99.2 | 333.6 | 8,05 | 7,81 | 23,6 |
|  | North Wyke | 158.8 | 451.8 | 628.5 | 993.4 | 1675.0 | 2442.9 | 1200.4 | 681.1 | 446.3 | 1943.5 | 7,11 | 7,61 | 31,3 |
|  | Hillsborough | -0.5 | 14.2 | 17.2 | 27.2 | 22.4 | 39.3 | 26.4 | 37.0 | 5.4 | 59.6 | 11,01 | 12,72 | 26,7 |
|  | Pwllperian | 34.5 | 136.5 | 237.5 | 324.2 | 613.8 | 628.2 | 295.6 | 356.6 | 60.5 | 843.1 | 6,86 | 7,60 | 63,2 |
| **3** | Crichton | 18.0 | 114.6 | 259.3 | 512.2 | 699.0 | 857.9 | 457.5 | 281.4 | 220.7 | 772.8 | 11,47 | 12,68 | 103,7 |
|  | Drayton | 0.0 | 29.6 | 89.9 | 102.8 | 126.6 | 172.5 | 88.2 | 216.6 | 100.2 | 151.1 | 13,98 | 16,09 | 26,6 |
|  | North Wyke | 89.9 | 273.4 | 303.4 | 747.0 | 780.0 | 1162.4 | 897.9 | 463.0 | 332.5 | 846.0 | 12,93 | 15,34 | 27,8 |
|  | Hillsborough | 7.8 | 15.9 | 23.8 | 72.6 | 73.8 | 129.4 | 50.5 | 77.9 | 9.6 | 95.0 | 10,74 | 13,27 | 48,2 |
|  | Pwllperian | 13.0 | 314.3 | 466.1 | 641.7 | 911.4 | 886.0 | 485.6 | 682.4 | 64.8 | 947.0 | 11,46 | 14,67 | 100,9 |
| **4** | Crichton | 69.0 | 156.3 | 382.2 | 788.5 | 1291.4 | 2281.3 | 674.5 | 1222.2 | 465.4 | 1763.3 | 15,17 | 16,88 | 96,6 |
|  | Drayton | 25.8 | 61.8 | 72.2 | 108.4 | 120.8 | 114.2 | 95.9 | 79.9 | 63.4 | 98.8 | 19,26 | 20,75 | 61,2 |
|  | North Wyke | 120.7 | 64.0 | 34.7 | 60.8 | 89.5 | 88.9 | 62.1 | 53.2 | 45.7 | 96.6 | 16,90 | 19,00 | 2,6 |
|  | Hillsborough | 11.9 | 106.5 | 90.9 | 138.1 | 163.6 | 248.2 | 138.5 | 288.8 | 56.0 | 132.6 | 13,64 | 15,53 | 50,2 |
|  | Pwllperian | 13.0 | 39.4 | 128.9 | 199.1 | 222.1 | 366.2 | 158.7 | 320.1 | 41.5 | 233.2 | 17,69 | 20,58 | 92,1 |
